# Variation in mortality burden of the COVID-19 pandemic across federal states in Germany

**DOI:** 10.1093/eurpub/ckad110

**Published:** 2023-07-20

**Authors:** Marina Kolobova, Dmitri Jdanov, Domantas Jasilionis, Vladimir M Shkolnikov, Roland Rau

**Affiliations:** Max Planck Institute for Demographic Research, Rostock, Germany; Max Planck Institute for Demographic Research, Rostock, Germany; International Laboratory for Population and Health, National Research University Higher School of Economics, Moscow, Russia; Max Planck Institute for Demographic Research, Rostock, Germany; Vytautas Magnus University, Kaunas, Lithuania; Max Planck Institute for Demographic Research, Rostock, Germany; International Laboratory for Population and Health, National Research University Higher School of Economics, Moscow, Russia; Max Planck Institute for Demographic Research, Rostock, Germany; University of Rostock, Rostock, Germany

## Abstract

**Background:**

Intra-annual excess mortality is the most reliable measure of losses of lives due to short-term risk factors. The objectives of our study are (i) to estimate excess mortality across German states in the course of the coronavirus disease 2019 (COVID-19) pandemic years 2020 and 2021 and (ii) to identify possible regional-level determinants of spatial inequality in pandemic-related excess mortality.

**Methods:**

We use weekly mortality data series for the calculation of weekly death rates, standardized by age for each federal state of Germany. We estimate the expected level of mortality as state-specific mortality trends and excess mortality in 2020 and 2021. We explore ecological statistical relationships between excess mortality, COVID-19 morbidity, and selected regional socioeconomic indicators using fixed-effects regression models.

**Results:**

Our study shows that during the first pandemic year, there was South-to-North gradient in excess mortality in Germany, with excess mortality being higher in the South. Over the course of the second pandemic year 2021, this gradient changed to become an East-to-West gradient, with excess mortality being higher in the East. The results of the study show stronger effects of COVID-19 morbidity on excess mortality in East Germany. State-level indicators reflecting economic activity, employment, and capacity of intensive care units show significant correlations with excess mortality across the states.

**Conclusions:**

The results show pronounced state-level differences in the magnitude of excess mortality during the COVID-19 pandemic in Germany. Economic activity, employment and capacity of intensive care units were the most important state-level characteristics associated with the observed spatial variations in excess mortality.

## Introduction

The onset of the coronavirus disease 2019 (COVID-19) pandemic created new challenges for public health and mortality research. The numbers of confirmed disease-related cases and deaths are likely to be underestimated, as they are dependent on COVID-19 surveillance. Compared with the case fatality indicator, the estimation of all-cause excess mortality provides a more precise and complete picture of the real mortality burden of the pandemic.^1^

Much before the pandemic, persisting regional mortality disparities within countries were considered an important challenge for policymakers seeking to achieve sustainable mortality improvements. Therefore, it is not surprising that regional differentials became even more important during the pandemic period.

Cross-country studies at the subnational level have uncovered changing temporal and spatial patterns of excess mortality localizations across the regions of European countries during the first and the second pandemic waves.^2^ A North-to-South gradient in excess mortality has been found for Italy, with the northern regions of Italy having much higher excess mortality than the central and southern regions.^3^ In Spain, most excess deaths occurred in the central region.^4^ The extent of excess mortality and COVID-19 incidence varied by the socioeconomic status of the regions.^5^ Finally, rural–urban differences in excess mortality have been observed.^6^

Various studies have focused on excess mortality in Germany at both the national and the subnational levels. Zur Nieden et al. studied excess mortality in Germany (until week 20 of 2020) using the mean mortality of 2016–19 as a baseline mortality level.^7^ Stang et al. studied excess mortality in Germany during the first COVID-19 wave (from week 10 to 23 of 2020).^8^ They calculated excess mortality using the age-specific mean death rates for the period from 2016 to 2019 in Germany as the death rates of the standard population. Unexpectedly, the estimated number of excess deaths was found to be lower than the number of confirmed COVID-19-related deaths in Germany for the same period (8071 excess deaths versus 8674 COVID-19-related deaths). Nevertheless, König et al. showed that in Germany, excess mortality exceeded mortality related directly to COVID-19 both in 2020 and 2021.^9^^,^^10^ Böttcher et al. and Kowall et al. found no excess mortality in Germany in 2020.^11^^,^^12^ De Nicola et al. observed an overall excess mortality rate of 1% in 2020.^13^

Morfeld et al. used the arithmetic and geometric mean values of death numbers for the period from 2016 to 2019 as the expected mortality level for the calculation of the ‘simplified mortality ratios’ to show the regional mortality differences in Germany at the federal state level.^14^ Standardization on the temporal scale conducted by Morfeld et al.^14^ provided no insights into spatial mortality variation within Germany. It has also been demonstrated that the use of the mean mortality level is challenging if years with elevated mortality are included in the calculation of the baseline level.^15^ The same study suggested that substantial variability or even disagreement (as in the case of Germany) in excess mortality estimates produced by different authors may be related to variability in mortality indices, reference periods and definition of the baseline.

The main objective of the study is to provide comprehensive state-level estimates of the total mortality burden of the COVID-19 pandemic in Germany. Our study extends prior research on Germany in several ways. To our knowledge, this is the first study on Germany that covers 2 years of the COVID-19 pandemic. Moreover, in contrast to the previous research on excess mortality in Germany,^7–14^ we consider state- and week-specific mortality trends and use the forecasted values to estimate the expected level of mortality in 2020–21. We believe that such a methodological approach leads to a more complete and objective assessment of pandemic-related losses.

In addition, taking into account that differences in age composition may explain a part of the spatial variation in excess mortality,^16^ we apply an age-standardization procedure. The second objective of this study is to identify possible regional-level determinants of spatial inequality in pandemic-related excess mortality.

## Methods

For the calculation of mortality indicators, we use weekly and annual mortality data, and the population exposures from the Federal Statistical Office of Germany.^17^^,^^18^ The weekly mortality data series cover the period from 2000 to 2021. Weekly death counts for the whole of Germany are available by 5-year age groups for ages from 30 to 94, broad age group below 30 and open-ended age interval 95+ years. At the federal state level, these data are available by broader age groups only: 0–64, 65–74, 75–84, and 85+ years. Annual mortality data for German federal states are provided by 5-year age groups.

The numbers of confirmed COVID-19 cases per 100 000 inhabitants are published by the Robert Koch Institute (RKI) in the regularly issued situational reports starting from 4 March 2020 (RKI 2020).^19^ Data on the occupation of intensive care units (ICU) by COVID-19 patients are provided by the DIVI-Intensivregister of the RKI.^20^

To explore possible determinants of the spatial inequality in excess mortality, we use the socioeconomic indicators from the INKAR database.^21^ The INKAR offers up-to-date statistical information on socioeconomic development of German regions. Around 600 indicators present the current status as of 31 December 2020. Indicators reflecting socioeconomic composition can be divided into covariates reflecting average income level, educational composition of the employed population, unemployment and long-term unemployment, and occupation structure of the employed population. In addition, we selected covariates reflecting supply of the medical care in the region (e.g. distance to general practitioner, number of hospital beds per 1000 population), population and settlement density. We tested all available socioeconomic characteristics of the adult population and consider only the covariates showing statistically significant associations with excess mortality (see description of the covariates in Supplementary table SA1).

We calculate crude death rates (CDRs) for each week separately for females, males and both sexes. To estimate the age-standardized death rates (SDRs), we use the method proposed by Klimkin et al.^22^ This method transforms CDRs into SDRs using an adjustment coefficient. We calculate the adjustment coefficient using annual mortality data by 5-year age groups.


(1) $$R_{y}^{i}=\frac{\sum_{x}p_{x}^{s}\cdot M_{y,x}^{i}}{{\mathrm{CDR}}_{y}^{i}},$$

where ${\mathrm{CDR}}_{y}^{i}$ is the annual CDR in the region i in the year y, $M_{y,x}^{i}$ is the year-age-specific death rate in the region i in the year y in the 5-year age group x and $p_{x}^{s}$ is the age-specific population share in the standard population s. We use the population structure of Germany in 2020 as a standard population. Thus, the adjustment coefficient is a ratio of the annual SDR to the CDR.

We use the adjustment coefficient calculated for 2020 for the calculation of the weekly SDRs for the years 2020 and 2021:


(2) $${\mathrm{SDR}}_{y,w}^{i}={\mathrm{CDR}}_{y,w}^{i}\cdot R_{y}^{i},$$

where ${\mathrm{CDR}}_{y,w}^{i}$ is the CDR in the region i in the week w of the year y, and $R_{y}^{i}$ is the adjustment coefficient for the region i in the year y.

We estimate the expected mortality level using week-, sex- and state-specific mortality trends. The reference period for the estimation of the expected mortality level includes the period from 2000 to 2019 and excludes the years with elevated mortality due to influenza epidemics. In Germany (and in most European countries), the years with the highest winter mortality peaks and elevated influenza mortality were 2015, 2017 and 2018.^23^ Following Németh et al.,^24^ we calculate state-specific mortality trends separately for each week. The estimation consists of the set of independent week-specific OLS regression models:


(3) $$x_{i}=\hat{a_{i}}+\hat{b_{i}}T,$$

where $x_{i}$ is the estimated value of the mortality trend (e.g. the SDR), $\hat{a_{i}}$ and $\hat{b_{i}}$ are estimated coefficients of the OLS regression over the reference period for the week i and T is the target year.

We calculate the age-standardized intra-annual excess mortality as the difference between expected and observed levels of mortality:


(4) $${\mathrm{SDR}}_{\mathrm{excess}}={\mathrm{SDR}}_{\mathrm{observed}}-{\mathrm{SDR}}_{\mathrm{expected}},$$

where ${\mathrm{SDR}}_{\mathrm{expected}}$ is calculated using formulae (1)–(3) and ${\mathrm{SDR}}_{\mathrm{observed}}$ is calculated using formulae (1)–(2).

We study associations between weekly excess mortality and weekly COVID-19 morbidity using Pearson’s correlation coefficients with a time lag of 2 weeks. We chose the 2-week lag, because, depending on the virus variant, external conditions, the average time from symptom onset to death varies between 2 and 3 weeks. We also tested the 1-week lag and got similar results.

The statistical relationships between age-standardized excess mortality, COVID-19 morbidity and selected socioeconomic indicators are investigated using the fixed-effects regression models. State-specific cumulative numbers of excess mortality per 100 000 person-years standardized by age are used as dependent variables; while cumulative numbers of confirmed COVID-19 cases per 100 000 population, the percentage of the maximum occupation of the ICU and socioeconomic indicators are used as independent variables. We include East–West indicator in the models to account for persisting mortality differences between the two parts of Germany (former German Democratic Republic and Federal Republic of Germany). The effect of COVID-19 morbidity and socioeconomic indicators on excess mortality is modelled, as shown in the following equation:


(5) $$y=a+b_{1}x+b_{2}Z+\varepsilon ,$$

where y is the dependent variable (excess deaths per 100 000 person-years), x is an independent variable (COVID-19 morbidity or socioeconomic indicator), Z is an indicator variable (East–West) and ε is an error term. The models for each independent variable are estimated separately.

For the multivariate regression models, we tested all possible combinations of (significant) socioeconomic indicators, i.e. combinations that do not include correlated indicators, and selected the model with the highest explanatory power. We also include in the multivariate model a variable on ‘Maximum occupation of ICU by COVID-19 patients’ but do not include the number of registered COVID-19 cases. First, the number of registered cases depends on testing coverage and is not consistent over time. Second, the effects of other covariates in comparison with the number of registered COVID-19 cases are minor.

We aggregate the numbers of excess deaths over a period of two years during the pandemic, i.e. between week 10 of 2020, which marks the first week of the pandemic in Germany,^25^ and week 52 of 2021.

## Results

**Figure 1:**
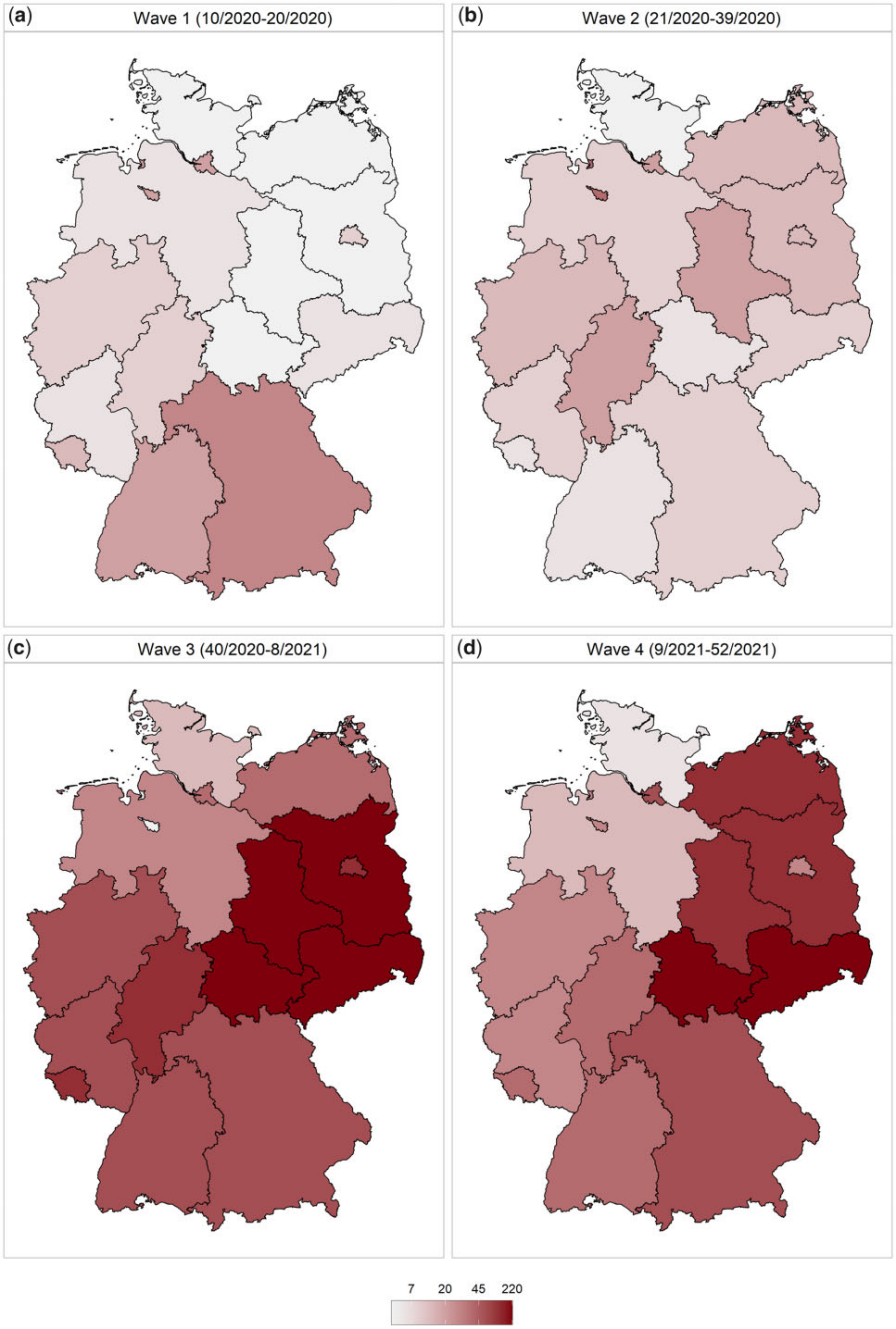
Cumulative numbers of age-standardized excess deaths per 100 000 person-years in Germany during the COVID-19 pandemic

Taking the state-specific mortality trends into account, we observe high levels of temporal and spatial heterogeneity in age-standardized excess mortality across the federal states of Germany. Figure 1 shows the cumulative numbers of estimated age-standardized excess deaths per 100 000 person-years during different periods of the COVID-19 pandemic.^25^ The southern federal states of Baden-Württemberg (19.6 excess deaths per 100 000 person-years) and Bavaria (22.6 excess deaths per 100 000 person-years), the city-states of Bremen (17.8 excess deaths per 100 000 person-years) and Hamburg (19.7 excess deaths per 100 000 person-years), had the highest levels of excess mortality during the first pandemic wave (figure 1a). This pattern changed over the course of the pandemic, with the South-to-North gradient in excess mortality turning into an East-to-West gradient, with the highest excess mortality being reported in the eastern federal states (figure 1c and d). The northern federal state of Schleswig-Holstein reported the lowest age-standardized excess mortality over the course of the pandemic (15.7 excess deaths per 100 000 person-years vs. 345.2 excess deaths per 100 000 person-years in Saxony).

**Figure 2:**
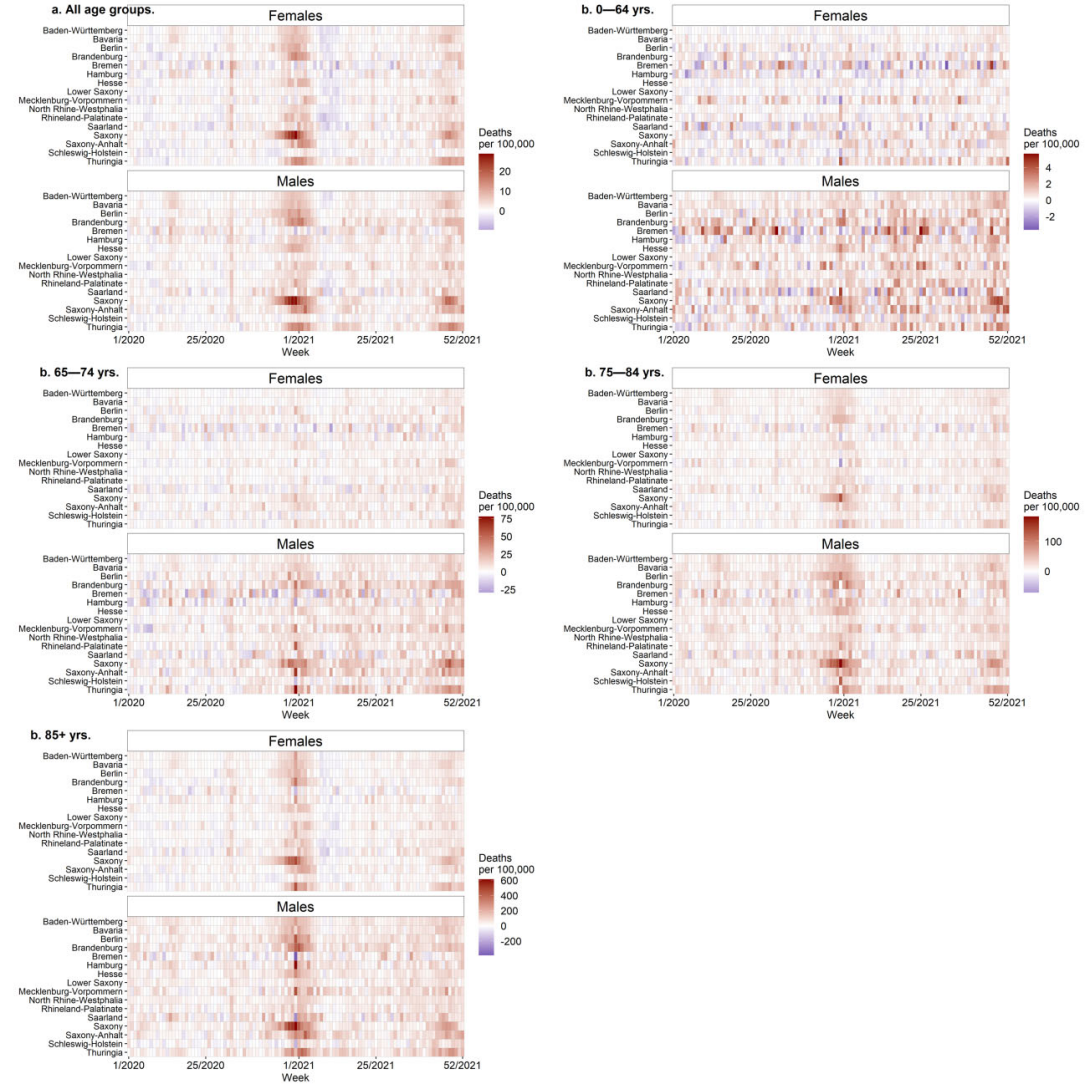
Age-standardized weekly excess deaths per 100 000 person-years in 2020–21 across federal states of Germany (a, females and males, all age groups; b, females and males, 0–64, 65–74, 75–84, and 85+ age groups)

Figure 2a and b shows weekly excess mortality by sex and age group during the first two pandemic years. An increase in excess mortality can be observed in all age groups at the end of 2020, at the beginning of 2021, and at the end of 2021. In Brandenburg, Saxony, Saxony-Anhalt and Thuringia, excess mortality increased much more dramatically than it did in other regions of Germany. Despite the observed heterogeneity, excess mortality was increasing steadily in most of the regions at the end of 2020. Females in the 64–75 age group experienced a shorter period of elevated excess mortality during this period that was concentrated in the south-eastern federal states. For both females and males, the lowest levels of excess mortality were experienced in the 0–64 age group.

In all age groups, males experienced higher levels of excess mortality than females. Consistent sex differences in excess mortality were found across all observed age groups and federal states of Germany.

**Figure 3:**
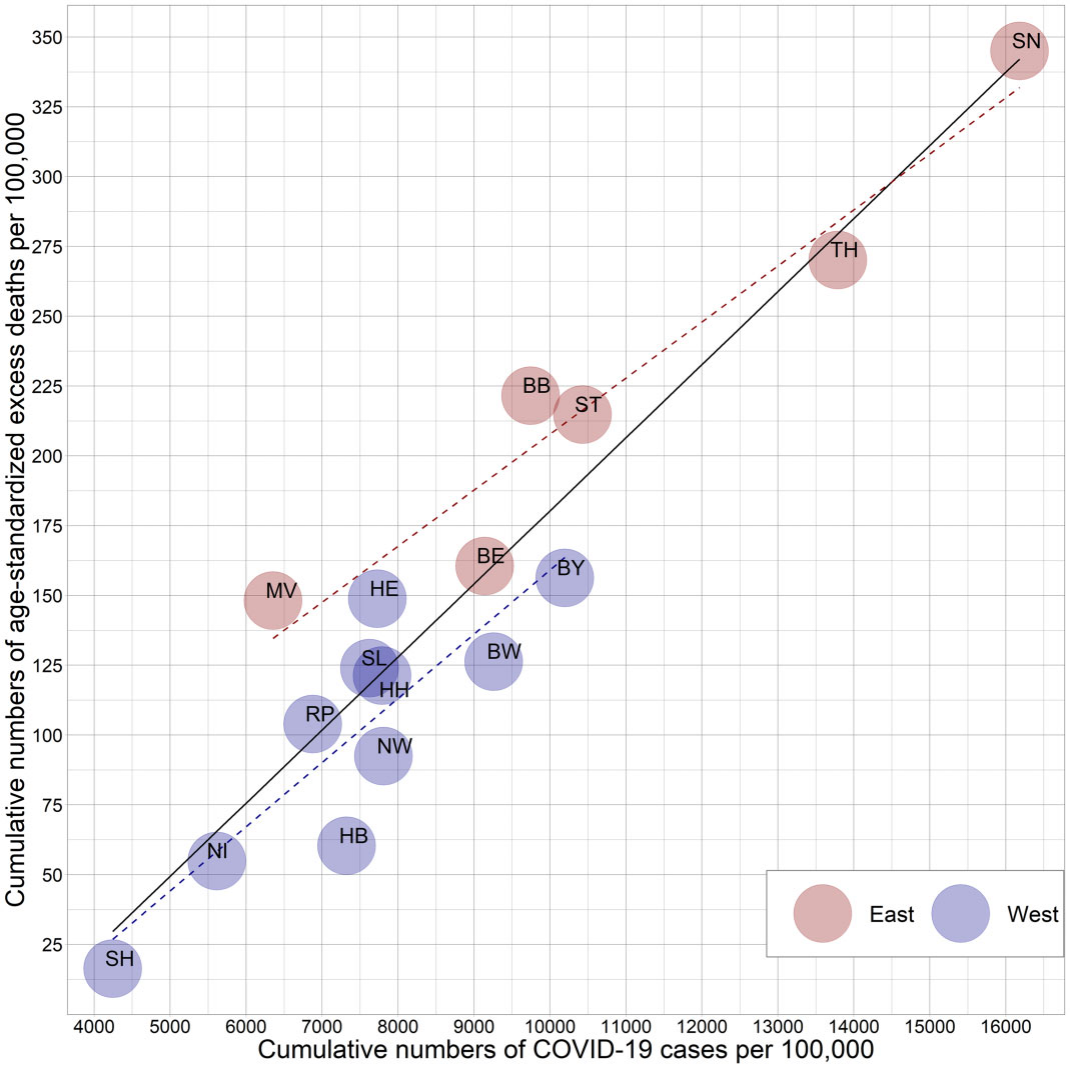
Association between cumulative numbers of excess deaths and COVID-19 cases per 100 000 population (week 10 of 2020 to week 52 of 2021)

Figure 3 shows the association between the age-standardized excess death rates and the numbers of confirmed COVID-19 cases per 100 000 population cumulated over the course of the pandemic in 2020 and 2021. Substantial differences between the federal states can be observed. The western federal states of Rhineland-Palatinate (104 604 excess deaths per 100 000 person-years) and Bremen (129.4 excess deaths per 100 000 person-years), which had higher COVID-19 morbidity, experienced lower excess mortality than the eastern federal state of Mecklenburg-Vorpommern (154.2 excess deaths per 100 000 person-years). The eastern federal states of Brandenburg (219.7 excess deaths per 100 000 person-years) and Saxony-Anhalt (211.1 excess deaths per 100 000 person-years) experienced higher excess mortality than the western federal state of Bavaria (163.6 excess deaths per 100 000 person-years), despite having the same level of COVID-19 morbidity. Two regional groups consisting of the eastern (except Berlin) and the western federal states can be distinguished. At the same level of registered COVID-19 morbidity, excess mortality was higher in the eastern than in the western part of Germany. Moreover, we observe a higher level of heterogeneity in the group of eastern federal states (standard deviation of 71.4 excess deaths per 100 000 person-years in the eastern federal states versus a standard deviation of 45.8 excess deaths per 100 000 person-years in the western federal states).

In the explanatory part of the study, we investigate the statistical relationships between excess mortality, COVID-19 morbidity and socioeconomic characteristics in the federal states. Table 1 presents the estimated regression coefficients of the fixed-effects regression models for the variables showing statistically significant results. As expected, COVID-19 morbidity indicators and maximum occupation of ICU by COVID-19 patients have a strong effect on excess mortality. We also observe that indicators reflecting the employment structure had a significant effect on excess mortality: i.e. the higher the employment rate, economic activity rate or employment rate of the population aged 55–65 was; the higher the excess mortality level was. In addition, we note that the results for the indicator of the educational structure of the employed population were quite unexpected, i.e. the higher the share of the employed population without vocational education was, the lower the excess mortality level was. The share of the employed population in atypical employment (part-time employment, and mini-jobs in employment) appeared to be negatively related to excess mortality at the federal-state level.

**Table 1:** Regression coefficients for independent fixed-effects regression models, including cumulative numbers of age-standardized excess deaths (over the period between week 10 of 2020 and week 52 of 2021) as the dependent variables, COVID-19 morbidity and selected structural indicators as the independent variables, and the East–West indicator as the group variable

Covariate	Regression coefficient
Cumulative numbers of confirmed COVID-19 cases	0.02 (0.02 to 0.03) Marginal *R*^2^/conditional *R*^2^ = 72.27%/91.25%
Maximum occupation of ICU by COVID-19 patients (percentage)	4.94 (2.1 to 8.92) Marginal *R*^2^/conditional *R*^2^ = 28.03%/69.81%
Employment rate	12.48 (4.76 to 22.03) Marginal *R*^2^/conditional *R*^2^ = 22.01%/71.13%
Economic activity rate (employed and unemployed population)	16.36 (3.75 to 28.81) Marginal *R*^2^/conditional *R*^2^ = 9.70%/79.25%
Employment rate of population aged 55–65 years	13.65 (3.97 to 25.81) Marginal *R*^2^/conditional *R*^2^ = 18.26%/68.55%
Part-time employment rate	−17.38 (−31.41 to −1.22) Marginal *R*^2^/conditional *R*^2^ = 7.59%/84.97%
Short-time employment rate	207.9 (20.76 to 392.05) Marginal *R*^2^/conditional *R*^2^ = 7.85%/77.21%
Share of mini-jobs in employment	−16.19 (−23.64 to −6.96) Marginal *R*^2^/conditional *R*^2^ = 54.39%/54.39%
Share of employed population without vocational education	−22.04 (−31.4 to −11.54) Marginal *R*^2^/conditional *R*^2^ = 58.44%/58.44%

Notes. 95% confidence intervals in the parentheses.

Supplementary table SA2 presents the estimated regression coefficients of the multivariate fixed-effects regression models. Model 4 has the highest explanatory power among tested models (see description of the regression analysis in the Methods). Maximum occupation in ICU by COVID-19 patients, employment rate and short-time employment rate has positive effect on excess mortality. In contrast, share of mini-jobs in employment shows negative correlation with excess mortality during the pandemic.

We also conducted additional analysis of the relationship between the weekly numbers of COVID-19 cases and the weekly numbers of excess deaths per 100 000 population for each federal state separately (Supplementary table SA3). As the results show, the weekly numbers of COVID-19 cases had no significant effect on the weekly excess mortality level for the northern federal states of Bremen, Hamburg, Lower Saxony and Schleswig-Holstein.

## Discussion

The first objective of our study was to thoroughly assess excess mortality across German states in the course of the COVID-19 pandemic years 2020 and 2021. The results revealed a strikingly high spatial variation in pandemic-related excess mortality even at such an aggregated level. The heterogeneity range from 15.7 age-standardized excess deaths per 100 000 population in the northern federal state of Schleswig-Holstein, to 345.2 age-standardised excess deaths per 100 000 population in the eastern federal state of Saxony.

We also observed the temporal change in the direction of the gradient. Our results confirm the findings of previous studies that there was a South-to-North gradient in excess mortality during the first year of the pandemic.^7^^,^^26^ However, this pattern changed over the course of the pandemic, with the South-to-North gradient turning into an East-to-West gradient in 2021. The study also observed some state-level associations between the reported COVID-19 morbidity and excess mortality. North-western federal states Bremen, Lower Saxony and Schleswig-Holstein had the lowest levels of COVID-19 morbidity and excess mortality, while South-Eastern states Saxony and Thuringia experienced the highest morbidity and excess mortality burden.

The second objective of the study was to identify possible state-level determinants of spatial inequality in pandemic-related excess mortality. The descriptive analyses suggest that eastern federal states had not only higher numbers of registered COVID-19 cases on average but also higher level of excess mortality by given morbidity. For example, over the course of the pandemic, the eastern federal states of Brandenburg, Mecklenburg-Vorpommern and Saxony-Anhalt had higher levels of excess mortality than the western federal states with similar levels of COVID-19 morbidity.

Our study also sheds some light on the sex-specific patterning of excess mortality. In an analysis of 10 European countries, Ahrenfeldt et al. (2021) found that men had a higher risk of COVID-19 mortality than women in almost all age groups.^27^ Bauer et al. showed that age dependency was stronger for COVID-19-related mortality than for all-cause mortality.^28^ Kolk et al. found that excess mortality in Sweden during the pandemic was concentrated at higher ages and was higher in men than in women.^29^ For Sweden, Modig et al. showed that while men had higher excess mortality than women up to age 75, excess mortality was similar among men and women at older ages.^30^ For Germany, we also found significant variation in excess mortality across age groups and men and women, with the highest excess mortality levels being observed in older age groups and in males.

In addition, our results show that COVID-19 morbidity had a strong effect on age-standardized excess mortality across the federal states of Germany. A higher ICU occupancy rate by COVID-19 patients was correlated with higher excess mortality, which indicates that ICU capacity and the preparedness of the health care system contributed substantially to the spatial variation in the burden of the COVID-19 pandemic.^31^^,^^32^ While the cumulative numbers of confirmed COVID-19 cases had a significant effect on the excess mortality cumulated over the study period, a correlation between weekly confirmed COVID-19 cases and weekly excess mortality was not observed for the northern federal states of Bremen, Hamburg, Lower Saxony and Schleswig-Holstein. This result may be explained by the differences in the testing strategies of the federal states.^33^

Another possible explanation for the East–West divide in excess mortality we found is that there were unobserved structural differences. We investigated the statistical relationships between age-standardized excess mortality and various indicators reflecting compositional and contextual characteristics at the subnational level. Various studies have confirmed the relationship between individual and area-level socioeconomic characteristics and COVID-19 morbidity, and mortality.^34–36^ However, of the socioeconomic indicators we considered, only a few were shown to have a significant effect on excess mortality. Differences in the employment structure were found to contribute to the explanation of the spatial variation in excess mortality in Germany. Prior area-level studies suggest the importance of socioeconomic characteristics such as low education and low income associated with higher excess mortality.^37–40^

Several limitations of this study should be acknowledged. First, it is important to note that most of the aforementioned studies examining area-level mortality inequalities and reporting significant area-level effects of socioeconomic disadvantages are based on a more detailed aggregation level (districts or counties). Meanwhile, our study using more aggregated state-level information does not find statistically significant correlations between socioeconomic characteristics (education and income) and state-level excess mortality. These associations should be tested in future studies using more detailed administrative unit data.

Second, although macro-level analysis can give certain insights into the inequalities in excess mortality across Germany, the reported ecological associations between aggregated state-level indicators should be interpreted with a caution. Thus, the results of our study reflect the regional structural differences in the context of the COVID-19 pandemic and do not provide any micro-level explanations. However, the macro-level analysis might obscure spatial differences in mortality, since life expectancy in Germany varies substantially across the federal states.

Third, due to limited data availability, our age-specific analysis has been conducted only for four age groups. Fourth, the analyses of the associations between the reported COVID-19 morbidity and excess mortality might be biased by the differences in testing coverage across federal states. Finally, the excess mortality approach may underestimate the total pandemic-related excess mortality burden during the lockdown and overestimate the influence of the pandemic if other short-term risk factors are presented at the same time.

Despite its limitations, the study provides comprehensive evidence about pronounced state-level differences in the magnitude of excess mortality during the COVID-19 pandemic in Germany. This is a surprising and worrying fact that substantial inequalities persisted even at such high aggregation level in a country with a strong social security, relatively low social inequality, and equitable and well-resourced health care system. Further research using more detailed and comprehensive data is needed in order to understand mechanisms and determinants of the pandemic-related mortality disparities across areas in Germany.

*Conflicts of interest*: None declared.

## Supplementary Material

ckad110_Supplementary_Data

## Data Availability

The mortality and population data for Germany and German federal states are publicly available and were obtained from the German Federal Statistical Office (www.destatis.de). South–North gradient in excess mortality in the first year of the pandemic was replaced by the East–West gradient in the second year. Higher excess mortality in the eastern than in the western federal states persisted at the same level of coronavirus disease 2019 morbidity. Capacity of the intensive care unit and employment structure of the subnational populations contributed to the observed differences in excess mortality across the states in Germany. Further research using more detailed and comprehensive data is needed to understand mechanisms and determinants of the pandemic-related mortality disparities across areas in Germany.
