# Supplementary material for: Variation in mortality burden of the COVID-19 pandemic across federal states in Germany
Source: Eur J Public Health. 2023 Jul 20;33(5):930–6. doi: 10.1093/eurpub/ckad110 (PMC10567244; doi:10.1093/eurpub/ckad110)
Supplement: ckad110_Supplementary_Data [file ckad110_supplementary_data.pdf]

Supplementary materials.

Table A-1: COVID-19 morbidity and socioeconomic indicators used in the regression analysis, their means and standard deviations.

Covariate	East		West	
	Mean	Standard deviation	Mean	Standard deviation
Cumulative numbers of confirmed COVID-19 cases, number per 100,000 population	10,940.83	3,508.19	7,447.2	1,672.08
Maximum occupation of ICU by COVID-19 patients, percentage	36.47	10.09	27.47	6.06
Employment rate, percentage of population aged 15-65 years	63.35	3.66	60.55	2.49
Economic activity rate, percentage of employed and unemployed population aged 15-65	84.22	2.64	84.4	1.6

years				
Employment rate of population aged 55-65, percentage of population aged 55-65 years	57.71	2.82	55.31	2.27
Part-time employment rate, percentage of employed population	30.66	1.9	28.66	1.68
Short-time employment rate, percentage of employed population	0.2	0.16	0.22	0.13
Share of mini-jobs in employment, percentage of employed population	12.49	0.63	19.34	1.98
Share of employed population without vocational education, percentage of employed population	7.89	1.67	13.09	0.93

Table A-2: Regression coefficients for fixed-effects multivariate regression models, including cumulative numbers of age-standardised excess deaths (over the period between week 10 of 2020 and week 52 of 2021) as the dependent variables, COVID-19 morbidity and selected structural indicators as the independent variables, and the East-West indicator as the group variable.

Covariate	Regression coefficient			
	Model 1	Model 2	Model 3	Model 4
Maximum occupation of ICU by COVID-19 patients (percentage)	4.32 (2.29, 7.15)	4.25 (2.6, 6.18)	3.97 (1.97, 5.98)	3.87 (2.49, 5.25)
Employment rate	10.87 (5.47, 17.79)	8.33 (3.7, 13.66)	10.74 (5.64, 15.85)	7.87 (4.08, 11.66)
Short-time employment rate	-	136.43 (32.65, 233.09)	-	160.04 (80.85, 239.24)
Share of mini-jobs in employment	-	-	-7.65 (-12.44, -2.85)	-9.5 (-12.92, -6.08)
Marginal R^2/Conditional R^2	59.73%/81.68%	56.78%/88%	85.47%/85.47%	92.41%/92.41%

Notes. 95% confidence intervals in the parentheses.

Table A-3: Correlation coefficients between excess mortality and COVID-19 morbidity for the federal states of Germany across weeks of 2020 and 2021.

Federal state	Excess deaths and number of COVID-19 cases (time lag of 1 weeks)		Excess deaths and number of COVID-19 cases (time lag of 2 weeks)	
	Pearson's correlation coefficient	P-value	Pearson's correlation coefficient	P-value
Baden-Württemberg	0.485 (0.316, 0.625)	P-value < 0.001	0.396 (0.212, 0.554)	P-value < 0.001
Bavaria	0.535 (0.374, 0.664)	P-value < 0.001	0.425 (0.244, 0.577)	P-value < 0.001
Berlin	0.452 (0.276, 0.598)	P-value < 0.001	0.462 (0.287, 0.607)	P-value < 0.001
Brandenburg	0.558 (0.402, 0.682)	P-value < 0.001	0.45 (0.273, 0.597)	P-value < 0.001
Bremen	0.062 (-0.14, 0.259)	P-value = 0.548	0.086 (-0.118, 0.282)	P-value = 0.409
Hamburg	0.125	P-value = 0.227	0.208	P-value < 0.005

	(-0.078, 0.317)		(0.006, 0.393)	
Hesse	0.467<br><br>(0.294, 0.61)	P-value < 0.001	0.4<br><br>(0.216, 0.557)	P-value < 0.001
Lower Saxony	0.224<br><br>(0.024, 0.406)	P-value < 0.05	0.067<br><br>(-0.136, 0.265)	P-value = 0.519
Mecklenburg-Vorpommern	0.389<br><br>(0.204, 0.546)	P-value < 0.001	0.24<br><br>(0.04, 0.421)	P-value < 0.05
North Rhine-Westphalia	0.352<br><br>(0.163, 0.516)	P-value < 0.001	0.257<br><br>(0.058, 0.436)	P-value < 0.05
Rhineland-Palatinate	0.374<br><br>(0.188, 0.535)	P-value < 0.001	0.257<br><br>(0.058, 0.436)	P-value < 0.05
Saarland	0.294<br><br>(0.099, 0.467)	P-value < 0.01	0.251<br><br>(0.052, 0.431)	P-value < 0.05
Saxony	0.654<br><br>(0.522, 0.755)	P-value < 0.001	0.564<br><br>(0.41, 0.688)	P-value < 0.001
Saxony-Anhalt	0.517	P-value < 0.001	0.34	P-value < 0.001

	(0.353, 0.65)		(0.151, 0.509)	
Schleswig-Holstein	0.18<br>(-0.021, 0.367)	P-value = 0.079	0.114<br>(-0.09, 0.309)	P-value = 0.271
Thuringia	0.609<br>(0.466, 0.722)	P-value < 0.001	0.487<br>(0.316, 0.627)	P-value < 0.001
